# miRNA Sequencing and Differential Analysis of Testes from 1-Year-Old and 3-Year-Old Kazakh Horses

**DOI:** 10.3390/biology15070569

**Published:** 2026-04-02

**Authors:** Qiuping Huang, Mingyue Wen, Liuxiang Wen, Qunchang Li, Yaqi Zeng, Jianwen Wang, Jun Meng, Wanlu Ren, Xinkui Yao

**Affiliations:** 1 College of Animal Science, Xinjiang Agricultural University, Urumqi 830052, China; 2 Xinjiang Key Laboratory of Equine Breeding and Exercise Physiology, Urumqi 830052, China

**Keywords:** Kazakh horse, DemiRNAs, testes, development, reproduction

## Abstract

This study aims to elucidate the miRNA regulatory mechanisms during the developmental process of Kazakh horse testes at 1 and 3 years of age. Through miRNA sequencing and bioinformatics analysis of testicular tissues from 1-year-old and 3-year-old horses, a developmentally stage-specific miRNA expression profile was constructed. A total of 1640 miRNAs were identified, among which 437 (380 up-regulated and 57 down-regulated) exhibited significant differential expression between the two age groups, including eca-miR-16, eca-miR-17, eca-miR-103, and eca-miR-199a-5p. Functional enrichment analysis revealed that the target genes of these differentially expressed miRNAs were primarily involved in key processes such as oxidative stress response, hormone receptor signaling regulation, and cytoskeletal remodeling, suggesting that testicular maturation depends on a complex post-transcriptional regulatory network. Further KEGG analysis revealed significant enrichment of classic reproductive signaling pathways, including PI3K/AKT, Wnt/β-catenin, Hippo, and TGF-β, indicating their synergistic roles in spermatocyte proliferation/differentiation and testicular homeostasis establishment. Although limited by a small sample size, this study elucidates the molecular mechanisms underlying male reproductive maturation in Kazakh horses at the post-transcriptional regulatory network level, providing preliminary theoretical support and potential markers for evaluating stallion reproductive performance and molecular breeding.

## Simple Summary

This study explores the microRNA-mediated regulatory mechanisms governing testicular development in Kazakh horses at different sexual maturation stages (1 and 3 years of age). By establishing a stage-specific microRNA expression profile, we identified several key regulators, such as eca-miR-16, eca-miR-17, and eca-miR-103, that are significantly associated with testicular maturation. Functional analysis suggests that these microRNAs coordinately regulate essential biological processes, including hormone signaling, cytoskeletal remodeling, and oxidative stress responses. Furthermore, key reproductive pathways, such as PI3K/AKT and Wnt/β-catenin, were identified as core components of the post-transcriptional network driving spermatocyte development and testicular homeostasis. These findings provide a comprehensive molecular landscape of male reproductive maturation in stallions, offering potential molecular markers to support breeding strategies and reproductive performance evaluation in the equine industry.

## 1. Introduction

The Kazakh horse is an ancient breed that has existed in its current habitat for approximately ten thousand years, making it one of the world’s oldest horse breeds. Its modern descendants still exhibit color characteristics similar to those of the ancient European wild horse [1]. As a versatile breed, the Kazakh horse is renowned for its stable genetic traits and robust tolerance to coarse fodder. It holds significant social value within traditional societies across China and neighboring nations, serving as a crucial animal in agricultural production [2].

The testes are the core organs of the male reproductive system, performing two critical physiological functions: spermatogenesis and androgen secretion [3,4]. They also regulate the entire reproductive axis through endocrine mechanisms, maintaining normal sexual behavior and fertility [5]. Testicular development is closely linked to reproductive performance. Transcriptomic analysis of testicular tissues from Kazakh horses at different ages revealed distinct gene expression patterns across developmental stages [6]. At 1 year of age, horses enter puberty and a period of rapid growth. Testes are small at this stage, with limited spermatogenic capacity [7]. By 3 years, sexual maturity is reached, and testes exhibit fully developed architecture with the capacity for high-yield, high-quality sperm production [8]. During this period, the testes undergo rapid growth, with somatic cell proliferation and differentiation supporting the development of spermatogonia into mature sperm [9,10]. Prior to maturation, the testes engage in complex cellular differentiation, hormonal regulation, and gene expression networks, laying the foundation for subsequent spermatogenesis and androgen synthesis [11,12].

As a crucial class of non-coding RNA molecules, miRNAs exert key epigenetic regulatory roles in mammalian testis development and spermatogenesis [13]. Studies indicate that RNA sequencing technology has been applied to evaluate the regulatory roles of miRNAs in mammary gland development and stress responses during early pregnancy in Kazakh horses. These miRNAs play vital roles in cellular differentiation, biological development, and physiological processes [14]. Epigenetic regulators such as miRNAs exert critical functions in pre-mature testicular development [15,16]. He et al. utilized Solexa sequencing to analyze differential miRNA expression patterns in testicular tissue before and after sexual maturation, further confirming their core regulatory function during pre-spermatogenic development [17]. As central epigenetic factors, miRNAs coordinate spermatogonial stem cell behavior, support cell function, and regulate hormonal responses by finely tuning gene expression networks during pre-spermatogenic development [18], ensuring the timely initiation of spermatogenesis.

In male mammals such as stallions, testes undergo complex structural and functional remodeling prior to somatic maturation. This process relies heavily on dynamic gene expression regulatory networks, with miRNAs serving as one of the core regulators within these networks. Although no systematic studies directly targeting stallion testicular miRNAs have been reported to date, cross-species evidence strongly supports their conserved functional mechanisms. Given the close association between testicular development and miRNAs, deciphering the miRNA expression network and its target gene functions during this stage is crucial for elucidating the molecular basis of reproductive performance formation in Kazakh horses. This study conducted miRNA sequencing and differential expression analysis on testicular tissues from 1-year-old and 3-year-old Kazakh horses, providing theoretical foundations and potential molecular markers for breeding superior stallions and enhancing reproductive performance.

## 2. Materials and Methods

### 2.1. Ethics Statement

The experimental procedures and protocol of this study were approved by the Animal Ethics Review Committee of Xinjiang Agricultural University (Approval No. 2025001).

### 2.2. Tissue Collection

The experimental animals in this study were reared under identical conditions. Testicular tissue was collected from eight healthy horses aged one year (*n* = 4) and three years (*n* = 4), selected from the Tacheng region of Xinjiang, all reared under identical conditions. Testicular tissue was obtained from each horse via veterinary surgery, involving excision of a portion from the left testicle. Specimens were immediately preserved in liquid nitrogen and 4% paraformaldehyde solution for subsequent analysis. All horses were housed individually within the same stable, fed a standardized diet of high-quality dry alfalfa and corn pellets, and provided with free access to water.

### 2.3. RNA Extraction and Quality Analysis

Frozen testicular tissue was ground into a fine powder and processed with TRIzol (Invitrogen, Carlsbad, CA, USA) reagent according to the manufacturer’s protocol. RNA contamination and degradation were assessed using a 1% agarose gel. RNA quantity and purity were monitored using a NanoPhotometer^®^ spectrophotometer (IMPLEN, Westlake Village, CA, USA). Subsequently, the Qubit^®^ RNA Detection Kit (Life Technologies, Carlsbad, CA, USA) was used for RNA concentration measurement with a Qubit^®^ 2.0 Fluorometer (Life Technologies, Carlsbad, CA, USA). Analysis was also performed using the Nano 6000 assay kit (Agilent Technologies, Santa Clara, CA, USA) on an Agilent Bioanalyzer 2100 system (Agilent Technologies, Santa Clara, CA, USA), yielding RIN values > 7.0. miRNA was used for RNA-seq and quantitative real-time fluorescent quantitative PCR (qRT-PCR) analysis.

### 2.4. Library Preparation and Small RNA Sequencing

The miRNA libraries were constructed from 3 µg of total RNA per sample. Eight libraries from eight horse testis samples were prepared for sequencing using the NEBNext^®^ Multiplex Small RNA Library Prep Set for Illumina^®^ (NEB, Ipswich, MA, USA). PCR products were detected and purified on an 8% polyacrylamide gel (100 V, 80 min). DNA fragments matching 140–160 bp were dissolved in 8 μL elution buffer. Finally, library quality was assessed using an Agilent Bioanalyzer 2100 system with a DNA High Sensitivity Chip. Libraries were sequenced on the Illumina HiSeq 2500 platform (Illumina, San Diego, CA, USA), generating 50 bp single-end reads.

### 2.5. Read Filtering and Mapping on the Horse Reference Genome

The 3′ adapter sequences were trimmed using the Cutadapt v1.2.2 program, and reads shorter than 18 or longer than 35 nucleotides were removed. Read quality filtering was performed using the FASTQ quality filter (FASTX-Toolkit v0.0.13.2 http://hannonlab.cshl.edu/fastx_toolkit/, accessed on 3 May 2025) to discard reads with a Phred score below 20 for at least 80% of bases. Cleaned reads were mapped to the horse genome EquCab2.0 (GCF_000002305.2) and ECAY using BWA (v0.7.17). Although more recent genome assemblies are currently available, EquCab2.0 was utilized to maintain consistency with our rigorously established laboratory pipeline, which remains robust and reliable for capturing highly conserved miRNA profiles. Non-unique alignments were discarded. Aligned reads overlapping known miRBase (version 19, http://www.mirbase.org, accessed on 3 May 2025) were then counted using HTSeq-count (part of the HTSeq framework, v0.5.3p3) in “merge” mode. Additionally, reads mapping to equine tRNAs, rRNAs, snoRNAs, and snRNAs in the Rfam RNA family database (http://rfam.sanger.ac.uk/, accessed on 3 May 2025) were removed. New miRNAs were predicted using mireap (https://github.com/liqb/mireap, accessed on 3 May 2025) based on their characteristic hairpin precursor structures, mature miRNA sequences, and minimum free energy.

### 2.6. Differential Expression Analysis of miRNAs

Based on Blast results from the miRBase database and mireap predictions, read counts were normalized using the DESeq2 R/Bioconductor package [19] to account for compositional bias in the sequencing libraries. miRNA differential expression (DE) was analyzed using the DESeq2 R/Bioconductor package. Thresholds of |log2 fold change| > 1 and padj < 0.05 were considered indicative of significant differences. Volcano plots and heatmaps of testis-expressed miRNAs were generated using the DESeq2 R package.

### 2.7. GO Annotation and KEGG Pathways

Target genes of the identified miRNAs were predicted using horse-specific homologous miRNA data from miRBase (version 19) combined with TargetScan/miRanda algorithms. Due to the high evolutionary conservation of core miRNAs across mammals and the developing status of equine functional annotations at the commencement of this study, conserved target genes across species were verified with human miRNA data to provide a more robust functional reference. Functional enrichment analysis (Gene Ontology (GO) and Kyoto Encyclopedia of Genes and Genomes (KEGG)) was performed on target genes using DAVID (https://davidbioinformatics.nih.gov/, accessed on 6 May 2025) with a false discovery rate (FDR) threshold of <0.05, visualized via the ggplot2 package (version 3.5.1) in R.

### 2.8. Validation of miRNA Sequencing Accuracy via Quantitative Real-Time Fluorescent PCR (qRT-PCR)

Total RNA was reverse transcribed into cDNA using the SweScript RT I First Strand cDNA Synthesis Kit (Servicebio, Wuhan, China) via the stem-loop method. Nine miRNAs were selected from the differentially expressed miRNAs for RT-qPCR validation. U6 small nuclear RNA (U6 snRNA) was chosen as the internal reference gene for normalization due to its stable expression across equine tissues. Primer sequences, including stem-loop RT primers, forward primers, and reverse primers, were designed using Primer 5, and the detailed primer information is provided in Supplementary Table S1. RT-PCR was performed using the 2 × Universal Blue SYBR Green qPCR Master Mix (Servicebio, Wuhan, China). All qRT-PCR reactions were performed with three biological replicates and three technical replicates. The relative expression levels were calculated using the 2^−ΔΔCt^ method.

## 3. Results

### 3.1. Sample Size for Sequencing

This study subjected raw sequencing data to rigorous quality control. As shown in Supplementary Table S2, the proportion of valid data exceeded 98.5% across all samples, with Q30 base coverage exceeding 93.8%, an N (unknown base) proportion approaching 0%, and normal GC content distribution (49.18–51.29%). The alignment rate of valid data against the reference genome ranged from 94.83% to 95.43%. These metrics indicate reliable sequencing data quality, meeting requirements for subsequent bioinformatics analysis.

### 3.2. Construction of miRNA Expression Profiles

**Figure 1:**
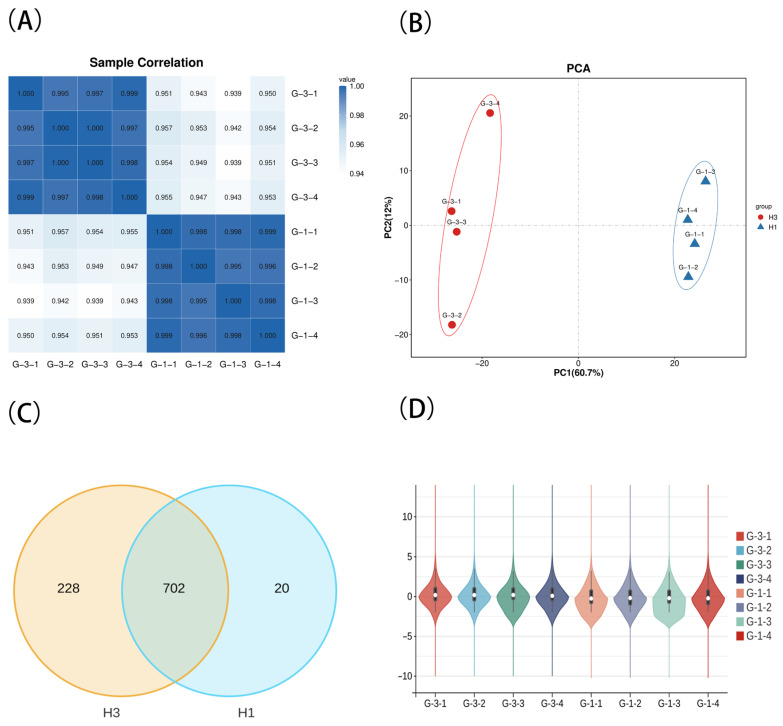
Sample relationship and expression distribution analysis. G-1 represents the 1-year-old sample; G-3 represents the 3-year-old sample. (**A**) Pearson correlation coefficient plot: Displays a heatmap illustrating sample correlations in descriptive expression. Color intensity indicates the strength of Pearson correlation coefficients (R^2^), ranging from light blue (0.94–1.0) to dark blue. The diagonal represents autocorrelation within the same sample (R^2^ = 1.0). (**B**) Principal Component Analysis (PCA) of horse testis tissue: X-axis (PC1) represents the first principal component—primary variation direction (60.7%); Y-axis (PC2) represents the second principal component—secondary variation direction (12%); (**C**) Venn diagram of identified miRNAs: Each colored ellipse represents an experimental group (H1, H3), using a threshold of TPM ≥ 1 to define expressed miRNAs. Overlapping areas indicate the number of commonly expressed miRNAs shared between groups, while non-overlapping regions denote group-specific miRNAs; (**D**) Violin plot: White dots denote the median; black rectangles span the interquartile range (IQR) from the first quartile (Q1) to the third quartile (Q3); Rectangle height represents the interquartile range (IQR). A longer IQR indicates more dispersed data, while a shorter IQR indicates more concentrated data. The black lines extending above and below represent the 1.5 × IQR confidence interval. Values outside these boundaries are considered outliers (<Q1 − 1.5 × IQR or >Q3 + 1.5 × IQR).

We performed miRNA sequencing on testicular samples from Kazakh horses aged 1 year (*n* = 4) and 3 years (*n* = 4). The results revealed distinct small RNA transcription profiles between the 1-year-old (H1) and 3-year-old (H3) samples. The findings indicate balanced sequencing quality across samples and effective data normalization, ensuring comparability for subsequent differential expression analysis (as shown in Figure 1).

### 3.3. Differentially Expressed MiRNAs

**Figure 2:**
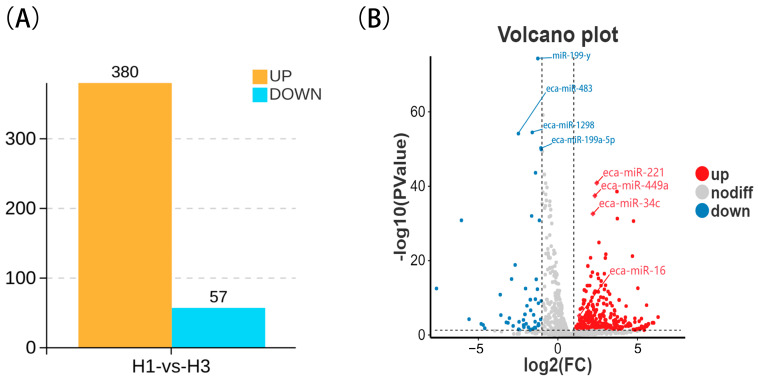
(**A**) Bar chart of significantly differentially expressed miRNAs (*p* < 0.05). (**B**) Volcano plot of differentially expressed miRNAs: The X-axis represents the log2 fold change (log2FC) in expression levels, and the Y-axis represents −log10 (*p*-value). Red dots indicate significantly upregulated miRNAs (log2FC > 1 and *p*-value < 0.05). Blue dots indicate significantly downregulated miRNAs (log2FC < −1 and *p*-value < 0.05). Gray dots represent non-significantly altered miRNAs.

To investigate the regulatory effects of miRNAs on testicular development in Kazakh horses, we compared and analyzed the miRNA expression profiles between the H1 and H3 groups. A total of 1640 miRNAs were identified, among which 437 exhibited significant differential expression. As shown in Figure 2 and Supplementary Table S3, after excluding non-significant gene results, 437 miRNAs exhibited significant differential expression (*p* < 0.05) between H1 and H3 groups in testicular tissue, including 202 unknown miRNAs. Among all significantly differentially expressed miRNAs, 380 were upregulated, including eca-miR-16, eca-miR-17, eca-miR-103, eca-miR-106a, and eca-miR-340-5p, while 57 were downregulated, such as eca-miR-199a-5p and eca-miR-211.

Among the significantly differentially expressed miRNAs, the specific expression level of eca-miR-16 increased from an average TPM of 125.042 in the H1 group to 318.334 in the H3 group (log2FC = 1.348, *p* < 0.05). Similarly, eca-miR-17 and eca-miR-103 also showed significant upregulation in the H3 group (log2FC = 1.299 and 1.403, respectively). Conversely, the downregulated eca-miR-199a-5p decreased significantly from an average TPM of 45,810.505 to 21,891.379 (log2FC = −1.065, *p* < 0.05). A comprehensive list of all significantly differentially expressed miRNAs, including their detailed expression values (TPM) and fold changes, is available in Supplementary Table S3.

**Figure 3:**
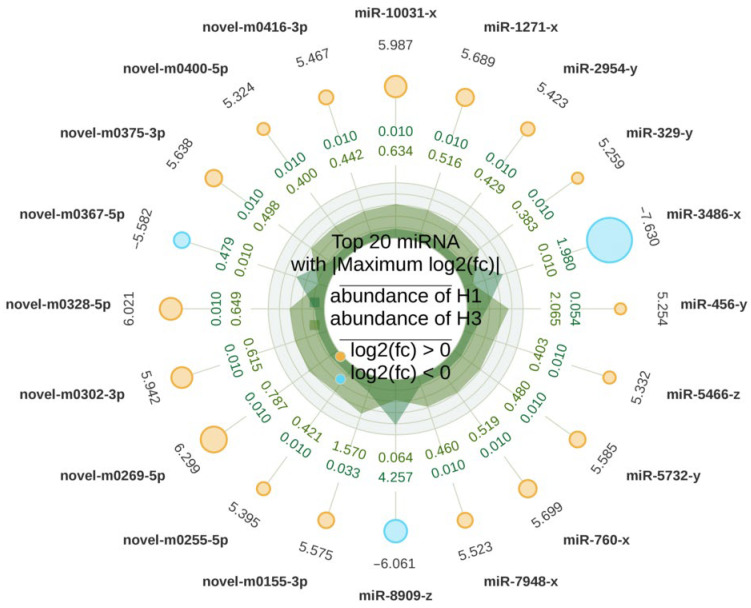
Radar plot of the top 20 most differentially expressed miRNAs. Outer ring values: log_2_(FC) values; Yellow sectors: Upregulated miRNAs (log2FC ≥ 0); Light blue sectors: Downregulated miRNAs (log2FC < 0); Sector area is proportional to |log2FC|. Third-layer double-layer heatmap: Outer ring shows average TPM for sample A; inner ring shows average TPM for sample B. Radial height reflects expression abundance. The central dot serves as a legend, with sample grouping labeled above and up/down regulation color-coded below.

The differentially expressed miRNAs were ranked, and the top 20 were selected. As shown in Figure 3, six of these miRNAs were unidentified. Among the known miRNAs, eca-miR-221, eca-miR-449a, eca-miR-34c, eca-miR-9014, and miR-8908-z were significantly upregulated, while miR-199-y, eca-miR-1298, eca-miR-483, eca-miR-199a-5p, miR-99-x, eca-miR-211, miR-199-x, miR-8909-z, and miR-300-y were significantly downregulated.

### 3.4. GO Enrichment Analysis of Differentially Expressed Genes

**Figure 4:**
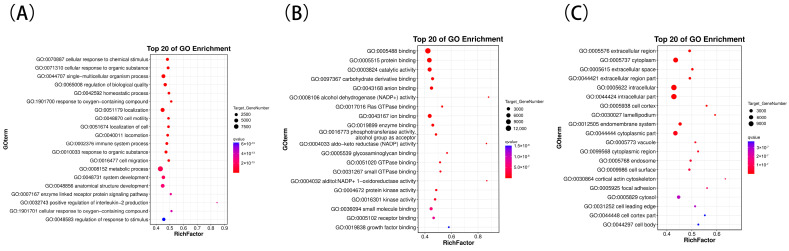
Level-2 Gene Ontology (GO) classification statistics for differentially expressed genes between G-1 and G-3. Differential gene screening criteria: |log_2_ FC| ≥ 1 and FDR ≤ 0.05 (**A**–**C**) represent the top 20 significantly enriched GO pathways for H1 vs. H3 differentially expressed genes in Biological Process, Molecular Function, and Cellular Component categories, respectively.

We performed GO enrichment analysis on significantly differentially expressed miRNAs between 1-year-old and 3-year-old Kazakh horses to characterize the functional and metabolic pathways of miRNA-regulated genes. Differentially expressed miRNAs in the H1 and H3 groups were primarily enriched in biological processes including regulation of response to stimulus (GO:0048583), cellular response to oxygen-containing compounds (GO:1901701), positive regulation of interleukin-2 production (GO:0032743), enzyme-linked receptor protein signaling pathway (GO:0007167), anatomical structure development (GO:0048856), system development (GO:0048731), and metabolic processes (GO:0008152). In terms of cellular composition, they are primarily enriched in components such as cell body (GO:0044297), cell cortex part (GO:0044448), and cell leading edge (GO:0031252). In terms of molecular function, they are primarily enriched in growth factor binding (GO:0019838), receptor binding (GO:0005102), small molecule binding (GO:0036094), kinase activity (GO:0016301), and protein kinase activity (GO:0004672) (as shown in Figure 4).

### 3.5. KEGG Pathway Analysis of Differentially Expressed Genes

**Figure 5:**
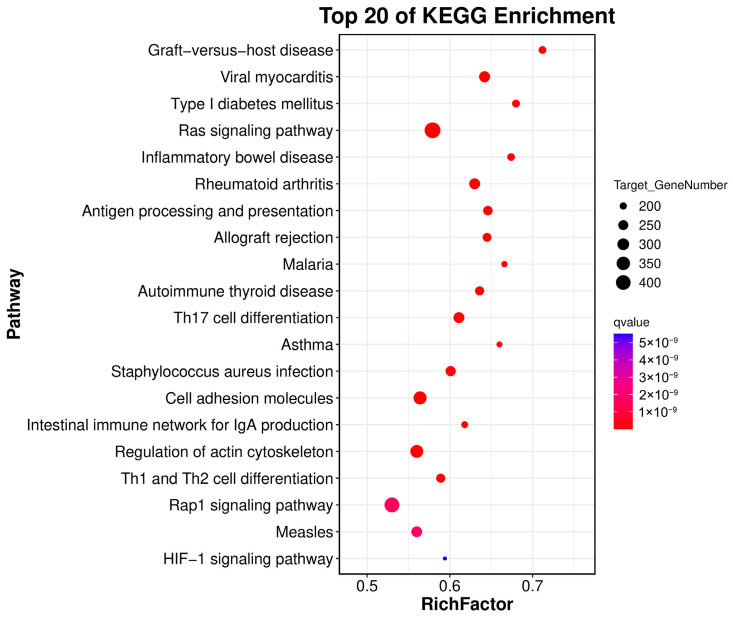
KEGG Enrichment Scatter Plot of H1 vs. H3 miRNAs (Top 20). The vertical axis represents different pathways, while the horizontal axis represents the proportion of significantly differentially expressed genes relative to all genes within that pathway. Circle size indicates the number of enriched genes in the pathway; larger circles denote greater enrichment. Color indicates enrichment significance; shades closer to purple indicate higher significance.

KEGG enrichment analysis of the target genes of significantly differentially expressed miRNAs in the testicles of 1-year-old and 3-year-old Kazakh horses (H1 and H3 groups) revealed primary enrichment in the following pathways: Rap1 signaling pathway, Th1 and Th2 cell differentiation, Regulation of actin cytoskeleton, Intestinal immune network for IgA production, Cell adhesion molecules, and Staphylococcus aureus infection (as shown in Figure 5).

### 3.6. RT-qPCR Validation of miRNA Sequencing Accuracy

**Figure 6:**
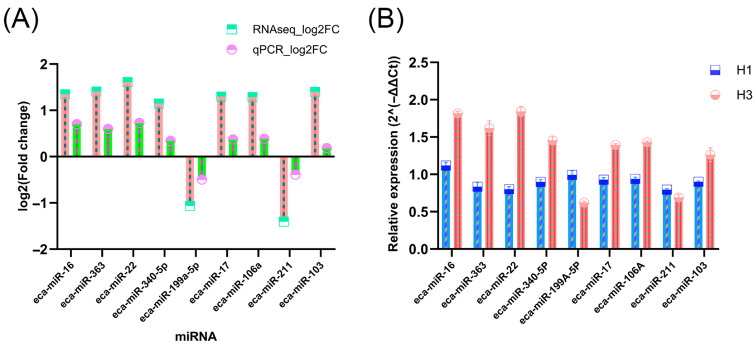
Validation of miRNA sequencing data using qRT-PCR. (**A**) Correlation of expression fold changes (log2FC) between RNA-seq and qRT-PCR for the nine selected miRNAs. The high linear correlation indicates the reliability of the RNA-seq quantification results. (**B**) Relative expression levels of the nine selected miRNAs between the H1 and H3 groups, determined by qRT-PCR (*p* < 0.05).

To validate the accuracy of RNA sequencing results, we performed qRT-PCR validation on nine selected miRNAs. As shown in Figure 6, all tested miRNAs exhibited expression patterns consistent with sequencing data. Seven miRNAs (eca-miR-16, eca-miR-22, eca-miR-17, eca-miR-103, eca-miR-106a, eca-miR-340-5p, and eca-miR-363) were upregulated, while two (eca-miR-199a-5p and eca-miR-211) were downregulated. To validate these expression changes, we determined the relative expression levels of nine miRNAs using RT-qPCR.

## 4. Discussion

As the core organ of the male reproductive system, testicular development relies on the precise temporal regulation of a series of key genes [11,20]. In recent years, miRNAs (miRNAs), as a crucial class of non-coding RNA molecules, have been increasingly revealed to play regulatory roles in germ cell differentiation. They serve as an important bridge linking gene expression regulation and organ development by post-transcriptionally modulating target gene expression, ensuring the timely initiation and orderly progression of spermatogenesis [18,21], and they target multiple critical biological processes involved in pre-mature testicular development [22]. In mammals, the miR-17-92 cluster is highly expressed in fetal testes and participates in regulating Sertoli cell proliferation and differentiation; its abnormal expression can lead to delayed gonadal development or structural abnormalities [23]. Furthermore, miR-202 exhibits stage-specific expression patterns in mouse testes, with significant upregulation during specific phases of the spermatogenic cycle, suggesting its potential role in temporally regulating spermatogenesis [24]. Similarly, miR-143 has been demonstrated to influence cell development and testosterone production [11]. More importantly, certain miRNAs affect gonadal differentiation toward the testis, directly participating in the regulatory networks of relevant genes [20]. This evidence indicates that miRNAs exist not merely as collateral regulatory elements but are deeply embedded within the core genetic programs governing testis development.

miR-16, as a highly conserved regulatory non-coding RNA, plays crucial roles in cell cycle regulation, proliferation suppression, and apoptosis induction. These functions are particularly critical during reproductive system development [25,26]. Studies indicate that in human and mouse models, miR-16 has been demonstrated to suppress tumor cell proliferation by targeting key signaling pathways such as IGF1R/Raf1-MEK1/2-ERK1/2, which also participates in regulating testicular interstitial cell function and androgen synthesis [27]. Furthermore, miR-16 has been found to influence angiogenesis by regulating VEGF expression, which is crucial for establishing the testicular microenvironment and forming the blood-testis barrier [25]. Existing research indicates that miR-16 is associated with various disease states, including cancer and cardiovascular disease, and it can transmit information between cells via exosomes [28,29]. Therefore, we hypothesize that the upregulation of eca-miR-16 in Kazakh horse testes may play a pivotal role in regulating testicular cell behavior and the local endocrine environment by targeting key developmental signaling pathways, rather than functioning as a viral component. Given the miR-16 family’s widespread role in cell proliferation, differentiation, and endocrine regulation, its differential expression is likely essential for modulating the homeostatic balance of the post-transcriptional regulatory network during testicular maturation. The miR-17–92 gene cluster constitutes a highly conserved polycistronic miRNA cluster playing pivotal roles in cell proliferation, apoptosis suppression, and tissue development, particularly serving as a core regulator in testis development and spermatogenesis [30]. Studies indicate that the miR-17–92 cluster is highly expressed in mouse testes, primarily localized in spermatogonial stem cells and Sertoli cells, where it contributes to maintaining male fertility. Knockout of this cluster leads to disorganized seminiferous tubule architecture, reduced spermatocyte numbers, impaired spermatogenesis, and ultimately oligospermia [30]. Therefore, in this study, the upregulation of eca-miR-17 may suppress key signaling pathway genes such as PTEN, RB1, or TGFBRII by targeting conserved 3’UTR sequences, thereby affecting testicular cell proliferation or differentiation processes. miR-103 has been demonstrated to promote polycystic ovary syndrome (PCOS) progression by targeting IRS1 and modulating the IRS1/PI3K/AKT signaling axis, which plays a central role in regulating the function of both ovarian granulosa cells and testicular Sertoli cells [31]. The PI3K/AKT signaling pathway not only regulates cell proliferation and survival but also participates in the initiation and maintenance of spermatogenesis. Furthermore, miR-103 promotes epithelial–mesenchymal transition (EMT) and cell migration in various cancers by regulating the Wnt/β-catenin pathway [32] and the Hippo pathway (e.g., by targeting LATS2) [33]. Notably, the Wnt and Hippo pathways are equally critical in early testicular developmental events such as embryonic gonadal differentiation, somatic cell fate determination, and blood–testis barrier formation [11]. Therefore, the upregulation of eca-miR-103 in this study may act as a regulator of testicular development by targeting core genes in key signaling pathways such as IRS1/PI3K/AKT, Wnt/β-catenin, or Hippo, thereby influencing Sertoli cell function, Leydig cell steroidogenesis, or the cyclical processes of spermatogenic cells. The expression levels of miR-199a-5p exhibit significant variations across various pathological states and developmental stages, suggesting its potential involvement in critical processes such as testicular cell proliferation, differentiation, and hormone synthesis [34,35]. In a heterotriploid crucian carp model, miR-199a-5p was found to regulate spermatogenesis at the post-transcriptional level by targeting the Tekt1 (tektin-1) gene. Tekt1 is an essential protein for sperm flagellar structural assembly, and its abnormal expression can lead to reduced sperm motility or even infertility. When eca-miR-199a-5p expression is downregulated, its inhibitory effect on Tekt1 weakens, thereby promoting the translation and accumulation of this protein. This, in turn, disrupts normal sperm flagellar assembly. Previous studies indicate that disrupted sperm flagellar assembly leads to both morphological defects and functional impairment [34]. Our finding regarding the significant downregulation of eca-miR-199a-5p is consistent with this pathway, suggesting its critical regulatory role in maintaining normal sperm integrity.

The oxidative stress response has been demonstrated to play a critical role in spermatocyte survival and DNA integrity maintenance [36,37], while IL-2 and related cytokine networks can influence Sertoli cell homeostasis and blood-testis barrier function. This suggests that differentially expressed miRNAs may promote testicular maturation by regulating immune homeostasis and oxidative balance [38]. Concurrently, enriched molecular functions such as growth factor binding, receptor binding, and protein kinase activity further indicate that miRNAs may profoundly participate in regulating growth factor receptors and their downstream kinase signaling. These signaling axes (e.g., IGF [39], TGF-β [40], VEGF [41], AKT [42]) have been confirmed as primary pathways modulating testicular development and spermatogenic function. Furthermore, the enrichment of differentially expressed miRNAs in cellular components like the cell body, cell leading edge, and cell cortex part, combined with cell migration and structural remodeling, provides the essential foundation for supporting the spatial rearrangement of supporting cells and Leydig cells during development [43,44]. Thus, miRNA-mediated post-transcriptional regulation permeates structural remodeling, signal transduction, and immune microenvironment modulation throughout development, serving as a key driver of testicular developmental timing. Further KEGG enrichment analysis reinforces this inference at the pathway level. Significant enrichment in the HIF-1 signaling pathway indicates central roles for oxygen homeostasis and energy metabolism regulation during stages H1 to H3. Previous studies demonstrate that HIF-1 promotes testicular vascularization and Leydig cell steroidogenesis in developmental hypoxia responses, while its dysregulation causes spermatogenesis impairment [45]. Additionally, differentially expressed miRNAs significantly enriched in Rap1 signaling, Regulation of actin cytoskeleton, and Cell adhesion molecules pathways collectively form the cell migration–adhesion–cytoskeletal remodeling axis. This axis is crucial for Sertoli cell polarization, gamete crossing of the blood-testis barrier, and spermatogonial migration to optimal microenvironments [38,46]. Concurrently, enrichment in Th1/Th2 cell differentiation and the Intestinal immune network for IgA production points to reproductive immune regulation. This aligns strongly with the recently proposed “testicular immune tolerance-selective immune activation” theory [47,48], suggesting miRNAs may maintain a delicate balance between immune protection and tissue remodeling demands. Notably, the presence of pathways associated with infection and stress (e.g., Measles, Staphylococcus aureus infection) does not necessarily indicate pathogen stimulation. Instead, it reflects the borrowing of innate and adaptive immune signaling modules during testicular development [49,50]. This cross-system signaling utilization has been confirmed in multiple studies on reproductive system development.

The integrated GO and KEGG enrichment results suggest that differentially expressed miRNAs do not act in isolation to influence single developmental events. Instead, they synergistically regulate testicular maturation through a four-tiered cascade network encompassing “stress response—growth factor signaling—immune regulation—cytoskeletal remodeling.” More importantly, multiple enriched pathways align closely with the target pathways of key miRNAs identified in this study (including eca-miR-16, eca-miR-17, eca-miR-103, eca-miR-199a-5p, and eca-miR-211), such as IGF1R, PI3K/AKT, Wnt/β-catenin, Hippo, and TGF-β signaling axes, further strengthening the causal link between differentially expressed miRNAs and testicular development mechanisms. This suggests that miRNA-mediated post-transcriptional reprogramming occurs during testicular development from H1 to H3. By synchronously regulating energy metabolism, cell migration, immune homeostasis, and endocrine function, this process ensures the establishment and maturation of spermatogenic capacity. While this study provides a comprehensive landscape of miRNA-mediated post-transcriptional reprogramming, we acknowledge that focusing solely on miRNA expression may not capture the full complexity of the regulatory networks. The integration of mRNA transcriptomics or proteomics data in future studies will be essential to validate these miRNA-target interactions and provide a more holistic understanding of the multi-layered regulatory mechanisms governing equine testicular development.

However, due to the limited availability and precious nature of Kazakh horse samples, the sample size in this study was relatively small. Additionally, Kazakh horses typically reach the appropriate age for castration only after 4 years of age, objectively limiting the acquisition of tissues from different developmental stages. These factors have, to some extent, affected the generalizability of the study results. Future work will expand the sample size, broaden the age gradient, and integrate multi-omics data to further validate and deepen the understanding of the key miRNAs and regulatory pathways identified in this study.

## 5. Conclusions

This study analyzed miRNA expression changes in the testicles of Kazakh horses at 1 and 3 years of age. Differentially expressed miRNAs were enriched in pathways including oxidative stress, immune regulation, growth factor signaling, and cytoskeletal remodeling, involving key signaling axes such as PI3K/AKT, Wnt, and Hippo. These findings suggest that testicular maturation in Kazakh horses depends on miRNA-mediated post-transcriptional regulatory reprogramming, with eca-miR-16, eca-miR-17, eca-miR-103, and eca-miR-199a-5p as core regulatory factors. Despite a limited sample size, this study provides important molecular evidence for the developmental mechanisms of equine testes.

## Data Availability

The data presented in this study are available upon request from the corresponding author.

## Supplementary Materials

The following supporting information can be downloaded at: https://www.mdpi.com/article/10.3390/biology15070569/s1, Table S1: Primer sequences used for qRT-PCR validation; Table S2: Summary of raw sequencing data and quality control metrics; Table S3: Differentially Expressed miRNAs.

## References

[1] Kusliy M.A., Yurlova A.A., Neumestova A.I., Vorobieva N.V., Gutorova N.V., Molodtseva A.S., Trifonov V.A., Popova K.O., Polosmak N.V., Molodin V.I. (2023). Genetic history of the Altai breed horses: From ancient times to modernity. Genes.

[2] Ilimkhanova L., Telebaev G., Dzakupov S., Perlenbetov M. (2012). The hierarchy of value orientations in Kazakhstan as the basis of national mentality. ҚазҰУ ХАБАРШЫСЫ.

[3] Li L., Lin W., Wang Z., Huang R., Xia H., Li Z., Deng J., Ye T., Huang Y., Yang Y. (2024). Hormone regulation in testicular development and function. Int. J. Mol. Sci..

[4] Gurung P., Yetiskul E., Jialal I. (2023). Physiology, male reproductive system. StatPearls [Internet].

[5] Morel M.C.D. (2015). Equine Reproductive Physiology, Breeding and Stud Management.

[6] Lu Z., Wen M., Yao X., Meng J., Wang J., Zeng Y., Li L., Ren W. (2025). Differential analysis of testicular LncRNA in Kazakh horses of different ages. Int. J. Biol. Macromol..

[7] Waqas M.-S., Arroyo E., Tibary A. (2024). Diagnostic Approach to Equine Testicular Disorders. Vet. Sci..

[8] Zhang F.L., Zhang X.Y., Zhao J.X., Zhu K.X., Liu S.Q., Zhang T., Sun Y.J., Wang J.J., Shen W. (2022). Multispecies comparative analysis reveals transcriptional specificity during Mongolian horse testicular development. Reprod. Domest. Anim..

[9] Stopel A., Lev C., Dahari S., Adibi O., Armon L., Gonen N. (2024). Towards a “testis in a dish”: Generation of mouse testicular organoids that recapitulate testis structure and expression profiles. Int. J. Biol. Sci..

[10] Su J., Yang Y., Wang D., Su H., Zhao F., Zhang C., Zhang M., Li X., He T., Li X. (2025). A dynamic transcriptional cell atlas of testes development after birth in Hu sheep. BMC Biol..

[11] Mäkelä J.-A., Koskenniemi J.J., Virtanen H.E., Toppari J. (2019). Testis development. Endocr. Rev..

[12] Dong P.-Y., Yan Y.-M.C., Chen Y., Bai Y., Li Y.-Y., Dong Y., Liu J., Zhang B.-Q., Klinger F.G., Chen M.-M. (2024). Multiple omics integration analysis reveals the regulatory effect of chitosan oligosaccharide on testicular development. Ecotoxicol. Environ. Saf..

[13] Wang L., Xu C. (2015). Role of microRNAs in mammalian spermatogenesis and testicular germ cell tumors. Reproduction.

[14] Liu L., Fang C., Sun Y., Liu W. (2021). Evaluation of key miRNAs during early pregnancy in Kazakh horse using RNA sequencing. PeerJ.

[15] La Y., Ma X., Bao P., Chu M., Guo X., Liang C., Yan P. (2023). Identification and profiling of microRNAs during yak’s testicular development. BMC Vet. Res..

[16] Hu R., Jiang X., Liu G., Chi S. (2022). Polygenic co-expression changes the testis growth, hormone secretion and spermatogenesis to prompt puberty in Hu sheep. Theriogenology.

[17] He L., Wang S., Deng H., Dong H., Chen J. (2018). Solexa profiling identifies differentially expressed miRNAs between sexually immature and mature equine testis. Braz. Arch. Biol. Technol..

[18] Luo Z.-Y., Dai X.-L., Ran X.-Q., Cen Y.-X., Niu X., Li S., Huang S.-H., Wang J.-F. (2018). Identification and profile of microRNAs in Xiang pig testes in four different ages detected by Solexa sequencing. Theriogenology.

[19] Love M.I., Huber W., Anders S. (2014). Moderated estimation of fold change and dispersion for RNA-seq data with DESeq2. Genome Biol..

[20] Sekido R., Lovell-Badge R. (2012). Genetic control of testis development. Sex. Dev..

[21] Liu Y., Sun Y., Li Y., Bai H., Xu S., Xu H., Ni A., Yang N., Chen J. (2018). Identification and differential expression of microRNAs in the testis of chicken with high and low sperm motility. Theriogenology.

[22] Han H., Chen Q., Gao Y., Li J., Li W., Dang R., Lei C. (2020). Comparative transcriptomics analysis of testicular miRNA from cryptorchid and normal horses. Animals.

[23] Fairchild M.J., Islam F., Tanentzapf G. (2017). Identification of genetic networks that act in the somatic cells of the testis to mediate the developmental program of spermatogenesis. PLoS Genet..

[24] Hu Y.-C., Namekawa S.H. (2015). Functional significance of the sex chromosomes during spermatogenesis. Reproduction.

[25] Nie Y., Zuo X., Xiong B., Ru K., Jia Y., Wang S., Yang R., Zhang D. (2017). Effect of Mir-16 Targeting VEGF on Angiogenesis of Myelodysplastic Syndromes. Blood.

[26] Yan X., Liang H., Deng T., Zhu K., Zhang S., Wang N., Jiang X., Wang X., Liu R., Zen K. (2013). The identification of novel targets of miR-16 and characterization of their biological functions in cancer cells. Mol. Cancer.

[27] Chen L., Wang Q., Wang G.-D., Wang H.-S., Huang Y., Liu X.-M., Cai X.-H. (2013). miR-16 inhibits cell proliferation by targeting IGF1R and the Raf1–MEK1/2–ERK1/2 pathway in osteosarcoma. FEBS Lett..

[28] Machtinger R., Laurent L.C., Baccarelli A.A. (2016). Extracellular vesicles: Roles in gamete maturation, fertilization and embryo implantation. Hum. Reprod. Update.

[29] Khalife J., Ghose J., Martella M., Viola D., Rocci A., Troadec E., Terrazas C., Satoskar A.R., Gunes E.G., Dona A. (2019). MiR-16 regulates crosstalk in NF-κB tolerogenic inflammatory signaling between myeloma cells and bone marrow macrophages. JCI Insight.

[30] Hurtado A., Palomino R., Georg I., Lao M., Real F.M., Carmona F.D., Burgos M., Jiménez R., Barrionuevo F.J. (2020). Deficiency of the onco-miRNA cluster, miR-106b~ 25, causes oligozoospermia and the cooperative action of miR-106b~ 25 and miR-17~ 92 is required to maintain male fertility. Mol. Hum. Reprod..

[31] Mu J., Yu P., Li Q. (2021). microRNA-103 contributes to progression of polycystic ovary syndrome through modulating the IRS1/PI3K/AKT signal axis. Arch. Med. Res..

[32] Zhao Y., Gu X., Wang Y. (2020). MicroRNA-103 promotes nasopharyngeal carcinoma through targeting TIMP-3 and the Wnt/β-catenin pathway. Laryngoscope.

[33] Han L.-L., Yin X.-R., Zhang S.-Q. (2018). miR-103 promotes the metastasis and EMT of hepatocellular carcinoma by directly inhibiting LATS2. Int. J. Oncol..

[34] Li S., Wang Q., Huang L., Fan S., Li T., Shu Y., Zhang C., Zhou Y., Liu Q., Luo K. (2022). miR-199-5p regulates spermiogenesis at the posttranscriptional level via targeting Tekt 1 in allotriploid crucian carp. J. Anim. Sci. Biotechnol..

[35] Shao S., Wang H., Shao W., Liu N. (2020). miR-199a-5p stimulates ovarian granulosa cell apoptosis in polycystic ovary syndrome. J. Mol. Endocrinol..

[36] Drobna M., Szarzyńska B., Jaksik R., Sędek Ł., Kuchmiy A., Taghon T., Van Vlierberghe P., Szczepański T., Witt M., Dawidowska M. (2020). hsa-miR-20b-5p and hsa-miR-363-3p affect expression of PTEN and BIM tumor suppressor genes and modulate survival of T-ALL cells in vitro. Cells.

[37] Kong X., Wang X., Xia Q., Hu Q., Yu W., Huang Q., Li J., Wang C., Lin Z., Liu Y. (2025). Unveiling the nexus between environmental exposures and testicular damages: Revelations from autophagy and oxidative stress imbalance. Cell Death Discov..

[38] Feng Y., Chen D., Wang T., Zhou J., Xu W., Xiong H., Bai R., Wu S., Li J., Li F. (2022). Sertoli cell survival and barrier function are regulated by miR-181c/d-Pafah1b1 axis during mammalian spermatogenesis. Cell. Mol. Life Sci..

[39] Ruan X., Jin X., Sun F., Pi J., Jinghu Y., Lin X., Zhang N., Chen G. (2024). IGF signaling pathway in bone and cartilage development, homeostasis, and disease. FASEB J..

[40] Young J.C., Wakitani S., Loveland K.L. (2015). TGF-β superfamily signaling in testis formation and early male germline development. Semin. Cell Dev. Biol..

[41] Tian R., Yang S., Zhu Y., Zou S., Li P., Wang J., Zhu Z., Huang Y., He Z., Li Z. (2016). VEGF/VEGFR2 signaling regulates germ cell proliferation in vitro and promotes mouse testicular regeneration in vivo. Cells Tissues Organs.

[42] Tsai Y.C., Kuo T.N., Chao Y.Y., Lee P.R., Lin R.C., Xiao X.Y., Huang B.M., Wang C.Y. (2023). PDGF-AA activates AKT and ERK signaling for testicular interstitial Leydig cell growth via primary cilia. J. Cell. Biochem..

[43] Kaltsas A. (2025). Multi-Omics Perspectives on Testicular Aging: Unraveling Germline Dysregulation, Niche Dysfunction, and Epigenetic Remodeling. Cells.

[44] Cortez J., Leiva B., Torres C.G., Parraguez V.H., De los Reyes M., Carrasco A., Peralta O.A. (2022). Generation and characterization of bovine testicular organoids derived from primary somatic cell populations. Animals.

[45] Shi Z., Hu C., Zheng X., Sun C., Li Q. (2024). Feedback loop between hypoxia and energy metabolic reprogramming aggravates the radioresistance of cancer cells. Exp. Hematol. Oncol..

[46] Nicholls P.K., Harrison C.A., Walton K.L., McLachlan R.I., O’Donnell L., Stanton P.G. (2011). Hormonal regulation of sertoli cell micro-RNAs at spermiation. Endocrinology.

[47] Mettelman R.C., Allen E.K., Thomas P.G. (2022). Mucosal immune responses to infection and vaccination in the respiratory tract. Immunity.

[48] Zhao S., Zhu W., Xue S., Han D. (2014). Testicular defense systems: Immune privilege and innate immunity. Cell. Mol. Immunol..

[49] Pradeu T., Thomma B.P., Girardin S.E., Lemaitre B. (2024). The conceptual foundations of innate immunity: Taking stock 30 years later. Immunity.

[50] Liu A., Garrett S., Hong W., Zhang J. (2024). Staphylococcus aureus infections and human intestinal microbiota. Pathogens.

